# Correlations between Psoriasis and Inflammatory Bowel Diseases

**DOI:** 10.1155/2013/983902

**Published:** 2013-07-21

**Authors:** Nevena Skroza, Ilaria Proietti, Riccardo Pampena, Giorgio La Viola, Nicoletta Bernardini, Francesca Nicolucci, Ersilia Tolino, Sara Zuber, Valentina Soccodato, Concetta Potenza

**Affiliations:** Department of Dermatology “Daniele Innocenzi”, A. Fiorini Hospital, Sapienza University of Rome, Via Firenze, Polo Pontino, 04019 Terracina, Italy

## Abstract

For a long time the relationship between inflammatory bowel diseases (IBDs) and psoriasis has been investigated by epidemiological studies. It is only starting from the 1990s that genetic and immunological aspects have been focused on. Psoriasis and IBD are strictly related inflammatory diseases. Skin and bowel represent, at the same time, barrier and connection between the inner and the outer sides of the body. The most important genetic correlations involve the chromosomal loci 6p22, 16q, 1p31, and 5q33 which map several genes involved in innate and adaptive immunity. The genetic background represents the substrate to the common immune processes involved in psoriasis and IBD. In the past, psoriasis and IBD were considered Th1-related disorders. Nowadays the role of new T cells populations has been highlighted. A key role is played by Th17 and T-regs cells as by the balance between these two cells types. New cytokines and T cells populations, as IL-17A, IL-22, and Th22 cells, could play an important pathogenetic role in psoriasis and IBD. The therapeutic overlaps further support the hypothesis of a common pathogenesis.

## 1. Introduction

For a long time the relationship between inflammatory bowel diseases (IBDs) and psoriasis has been investigated by epidemiological studies [1, 2]. It is only starting from the 1990s that genetic and immunological aspects shared by IBDs and psoriasis have been focused on. Nowadays, the use of common biological drugs confirms these findings and promotes the research in this area [3].

## 2. Epidemiological Correlations

The first epidemiological evidence from 1968 reported a prevalence of 2-3% of psoriasis in first-degree relatives of patients with Crohn's disease (CD) compared to 0–3% of controls; this association seemed to be less frequent for ulcerative colitis (UC) [4]. However a higher prevalence of psoriasis in patients with IBD was not reported. From the 1970s almost only case reports discussed the association between psoriasis and IBD, usually in the context of a third disease, such as oral lichen planus, thyroid adenoma, ankylosing spondylitis, the spondylitis associated with UC, Reiter's syndrome, and regional enteritis [5, 6].

The prevalence of psoriasis in the Caucasian population is 2-3%, and almost one-third of patients with psoriasis have a first-degree relative affected by the same disease [7]. This can be partially explained by inherited risk factors, as suggested by studies on concordance rates for psoriasis in monozygotic and dizygotic twins (70% versus 23%) [8].

The prevalence of CD in the general population is around 7 per 100,000 in the United States [9]. However, the risk for the siblings of a proband to be affected is 3%–5%. This confirms that CD, such as psoriasis, is much more common in families with affected members than in general population [10]. Concordance rates for Crohn's disease in monozygotic and dizygotic twins are 37% and 7%, respectively [11]. 

Families affected by Crohn's disease or psoriasis are also more likely to be affected by other immune-mediated diseases. 

Psoriasis and CD occur more often in the same person than it would be expected if the diseases were mutually exclusive. Data from five case-control studies reported a prevalence of psoriasis of 8.9% in patients with CD, but only of 1.4% in the control group (*P*< 1 × 10^−9^) [12].

Lee et al. in 1990 reported an incidence of psoriasis in patients with CD of 9.6%, while in the control group it was only 2.2% (*P* < 0.02), and incidence of psoriasis in relatives of patients with CD of 10%, compared to 2.9% in the control group (*P* < 0.02) [13]. 

A 2005 study on 8072 cases of IBD (3879 UC and 4193 CD) over a follow-up period of about 20 years showed a significant risk for both groups to have arthritis, asthma, bronchitis, psoriasis, and pericarditis if compared to controls. An increased risk of chronic renal failure and multiple sclerosis was observed in patients with UC, but not in those with CD [14]. 

One of the most recent papers about the epidemiologic association between IBD and psoriasis, conducted on 12502 psoriatic patients and 24287 controls, demonstrated that the prevalence of UC was significantly higher in patients with psoriasis compared to those of the control group, respectively 0.5% and 0.3%, with *P* = 0.002. Also the prevalence of CD was higher in patients with psoriasis compared to those of the control group, respectively 0.5% and 0.2%, with *P* = 0.001. In the same study multivariate analysis confirmed that psoriasis is associated with CD (OR: 2.49, 95% CI: 1.71 to 3.62) and UC (OR: 1.64, 95% CI: 1.15 to 2.33, and *P* = 0.006). This association remained statistically significant even after exclusion of patients treated with anti-TNF*α* drugs, respectively, with OR: 2.21%, 95% CI: 1.47 to 3.33, and *P* = 0.001 for CD and OR: 1.55, 95% CI: 1.08 to 2.22, and *P* = 0.017 for UC [15]. Consequently, a stronger association was found between psoriasis and CD than psoriasis and UC [16].

## 3. Genetic Correlations

**Figure 1:**
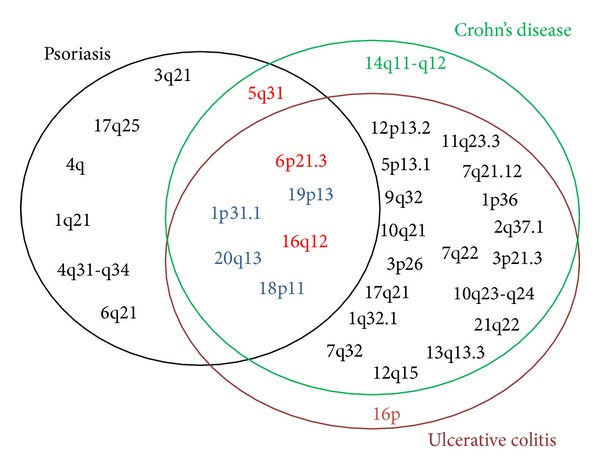


Several genetic correlations between psoriasis and IBD have been reported thanks to Genome Wide Association Studies (GWAS) that have identified 13 psoriasis susceptibility loci (called PSORS1-13) and 28 IBD susceptibility loci (called IBD1-28) (Figure 1). However, the pathogenetic relevance of each of these findings must be tested in an experimental setting.

The most important correlations involve the chromosomal loci 6p22, 16q, 1p31, and 5q33, and these associations will be analyzed in detail.

*Locus 6p22* (SNP: rs6908425) is located in the fifth intron of the *CDKAL1* gene (CDK5 regulatory subunit-associated protein 1-like 1) coding for a 65 kD protein with a still unknown function, which shares a domain with the protein CDK5RAP1 (CDK5 regulatory subunit-associated protein 1), an inhibitor of CDK5 (a protein kinase). The WTCCC study in 2007 demonstrated the association of this SNP (single nucleotide polymorphism) with CD [17]; later in 2008, Wolf et al. have found an association of 6p22 with psoriasis compared with healthy controls (OR: 1.26, 95% CI: 1.12 to 1.42, and *P* = 0.00015) [18]. This locus is associated with diabetes mellitus type II, too [19].

*Locus 16q* corresponds to PSORS8 and IBD3. Even if the association between psoriasis and IBD is well known, genes involving both conditions have not been found yet. *NOD2/CARD*15 gene (nucleotide binding oligomerization domain2/caspase-recruitment 15) is strongly related to CD but not to psoriasis. Concerning CD and NOD2/CARD15, three polymorphisms seem to be implicated [20]:

- R702W (C2104T), located between the NOD and LRR (Leucine-Rich Repeat) regions in exon 4;
- G908R (G2722C), located in the LRR region in exon 8;
- L1007P (3020insC), located in the LRR region in exon 11.

These polymorphisms are all defined as DCM (disease causing mutation) even if the first and the second ones are nonconservative missense mutations and do not produce obvious alterations of the protein structure as the 3020insC does, leading to synthesis of a truncated protein.

Heterozygosity for these simple allelic variants confers a slightly increased risk of developing CD (2 to 4 times than normal), while the state of homozygosity or compound heterozygosity increases the risk from 20 to 40 times [21]. A 2002 study [22] by Lesage et al. allowed further characterization of the pericentromeric region of chromosome 16 (where the *NOD2/CARD15* gene maps). Sixty-seven sequence variants, of which only 9 had an allele frequency greater than 5%, have been identified; among these, six polymorphisms were considered not associated with the disease, while the other 3 were confirmed to be independently associated with Crohn's disease susceptibility. Other 27 rare mutations were considered as potentially causing disease. The same paper reported an allelic frequency of the R702W, G908R, and L1007P mutations of 11%, 6%, and 11%, respectively. Concerning the DCMs (disease causing mutations) they represented the 32%, 18% and 31%. The other 27 DCMs mentioned earlier constituted the remaining 19%. This work also concluded that the great majority (93%) of the mutations were located in the distal third of the gene.

The NOD2 protein is expressed in antigen-presenting and epithelial cells [23] and seems to have a dichotomous function being involved in pro- and anti-inflammatory mucosal responses to microbial stimuli. This protein mediates the activation of nuclear factor *κ*B (NF-*κ*B), upon binding to muramyl dipeptide (MDP: minimum essential structure of the bacterial peptidoglycan).

Assuming that Crohn's disease is a consequence of an altered response of immune system to intestinal microflora components and that an excessive response to these components generates the inflammatory response, two different models can explain the association between the NOD2/CARD15 polymorphisms and Crohn's disease.

1. In the mucosa with a normal expression of nonmutated NOD2, the activation of TLR2 (toll-like receptor 2) induced by peptidoglycan of the intestinal flora and the consequent activation of nuclear factor NF-*κ*B are negatively regulated by the activation of NOD2 (a sort of negative feedback). Contrarily, in the mucosa where NOD2 is mutated the negative feedback is absent and the peptidoglycan is able to trigger a Th1 cell-mediated inflammation which leads to the development of Crohn's disease [24].
2. The second hypothesis argues that APCs (antigen presentation cells) resident in the lamina propria, called dendritic cells, use their dendrites and extend into the lumen of the mucosa, to incorporate bacteria and generate the ligand of NOD2 the MDP. The MDP spreads in Paneth cells and activates NOD2, that directly or indirectly induces the secretion of antimicrobial peptides known as *α*-defensins. So in the mucosa with a normal NOD2 expression the bacterial population is downregulated. On the contrary in the mucosa where NOD2 is mutated the lack of production of *α*-defensins leads to a bacterial overgrowth that induces the inflammatory response of the CD.

In psoriasis the link with the NOD2/CARD15 gene has been excluded [25].

An important contribution to this issue was provided by Zhu et al. that in 2012, in a meta-analysis of nine studies, confirmed the absence of association between psoriasis, psoriatic arthritis, and common polymorphisms of NOD2/CARD15; however the authors emphasized the importance of the protein encoded by this gene in the pathogenesis of psoriasis and psoriatic arthritis and the presence of conflicting results among the studies analyzed [26]. This conflict could be explained by several factors: primarily the genetic heterogeneity, as the NOD2/CARD15 polymorphisms vary considerably among the different ethnic groups, and secondly patients' age and disease duration [27]. On the basis of these elements Zhu et al. express a certain skepticism in definitively ruling out the association between psoriasis, psoriatic arthritis, and NOD2/CARD15.

*Locus 1p31.1*. The *IL-23R* gene maps onto this locus. The IL-23R has a fundamental pathogenetic role both in IBD (IBD17) and in psoriasis (PSORS7). For what concerns psoriasis in recent years 4 studies demonstrated the protective and predispositive action of two SNPs of this gene: rs7530511 (L310P) and rs11209026 (R381Q), respectively [28, 29]. In IBD risk variants different from psoriasis have been identified (rs7517847 and rs11805303), IBD and psoriasis share the same protective polymorphism, rs11209026 (R381Q), but different risk variants, rs7517847 and rs11805303. In addition, a protective role for V362I and G149R was assessed.

*Locus 5q33.1*. The association of psoriasis (PSORS11) and IBD (IBD19) with this locus is widely documented, especially with the polymorphisms of IL12B gene (i.e., IL-23B, or p40). For what concerns psoriasis the common alleles of the SNPs rs3212227 and rs6887695 have been identified as risk alleles. When taken together, these two alleles constitute a haplotype associated with psoriatic phenotype. In 2007 Capon et al. [30] confirmed these observations and identified common allele of the SNP rs1004431 as another risk allele. A protective role of all minor alleles of these SNPs, rs7709212, and p.Arg381Gln has been demonstrated. Moreover, in 2008 Nair et al. [31] found another SNP associated with psoriasis: rs2082412.

In IBDs the risk alleles identified were rs6556416 and rs6887695 that were found in psoriasis too.

Wolf et al. [18] discovered a polymorphism of the IRGM gene that maps onto the 5q33 locus (SNP: rs1000113), whose association with psoriasis, however, is still uncertain. This gene encodes a protein that contributes to the destruction of *Mycobacterium bovis* and belongs to the large family of IRG (immunity related GTPases), involved in the protection, and defense against bacterial pathogens. Given their importance, these genes (and the resulting proteins) are highly conserved during mammals evolution.

Finally, other less important pieces of evidence may be inferred from the analysis of the correspondences between different susceptibility loci reported for each of the diseases: 20q13 that corresponds to PSORS12 and IBD24, 19p13 that corresponds to PSORS6 and IBD6, 6p21 that corresponds to PSORS1 and IBD3, and 5q31 that corresponds to PSORS11 and IBD5 and 4q27 in which the IL-2 and IL-21 genes map [32]. According to these associations there is not a definitive evidence of mutual correspondence between psoriasis and IBD, often because the genes involved are different. Two loci appear to be more important thus needing further studies.

- 6p21, in which MHC classes I and II genes are found and whose correlation with psoriasis is consolidated since PSORS1, which is the main psoriasis susceptibility locus, maps onto 6p21.3. In this locus, the HLA-Cw∗0602 allele confers the greater risk [33], but this allele is present in only the 60–65% of affected individuals and can be found in 15% of the healthy population; only the 10% of the carriers will develop psoriasis [34]. To date there is still not a demonstration of the involvement of these genes in IBD, except for three SNPs: rs9268877, rs9268858, and rs9268480; the association with this locus appears to be mainly linked to polymorphisms in the TNF*α* gene promoter (308G → A polymorphism increases the risk for CD, 238G → A polymorphism reduces the levels of TNF*α* in patients with UC; −857C polymorphism increases the risk of IBD only in patients with the common mutations of NOD2/CARD15). Even the predisposition to psoriasis could be linked to polymorphisms in the TNF*α* genes promoter, such as −238G G → A polymorphism [35].
- 5q31: in this locus allelic variants for both psoriasis and IBD have been found [36, 37]. In psoriasis an association with three variants of the IL-13 gene was observed [38]: rs1800925, rs20541, and rs848 (This cytokine, such as IL-4 and IL-10, is secreted by Th2 lymphocytes and seems to be important in the innate and adaptive immune response dysregulation that leads to psoriasis.) A less strong association has been found with the rs1156806 variant of the SLC22A4 gene (OR: 0.68, 95% CI: 0.47 to 0.99, and *P* = 0.043), but by the combination of this SNP with the rs1800925 variant of the IL1-3 gene two common haplotypes strongly associated with psoriasis result. Finally there is a marginal association with psoriasis and different variants of a 370 kb region in which the IL-4, IL-5, and IRF-1 genes map. In IBD, there is a correlation with two risk haplotypes: IGR2198a_1 (rs11739135) and IGR2096a_1 (rs12521868), with the polymorphisms 1672C → T (L503F) of SLC22A4 (OCTN1) and −207 g → C of the SLC22A5 promoter (OCTN2) and with various polymorphisms of the P4HA2 and IRF-1 genes.

## 4. Pathogenetic Correlations

A lot has been written about molecular mechanisms involved in psoriasis and IBDs, but there are few studies trying to compare these mechanisms, in particular when these conditions affect the same patient at the same time. According to current knowledge, both psoriasis and IBD recognize two pathogenetic moments, the first one involving innate immunity triggered by unknown stimuli and the second one involving the adaptive immunity, due to cytokines released from cells of the innate immune system, mainly dendritic cells. Cytokines subsequently influence the activity of T cells subtypes as T-helper17 (Th17) and T-regs, now considered as crucial within the pathogenetic process.

### 4.1. T-Helper 17 Cells

Recently discovered, Th17 are a subpopulation of T-helper cells that produce mainly interleukin 17; they are considered evolutionarily distinct from Th1 and Th2 and play a key role in inflammatory processes and tissue damage in chronic conditions such as psoriasis, autoimmune uveitis, juvenile diabetes, rheumatoid arthritis, Crohn's disease, and multiple sclerosis [39, 40]. Th17 have also a very important function in antimicrobial immunity on epithelial and mucosal barriers through the production of cytokines, such as IL-22, which stimulate epithelial cells to synthetize antimicrobial proteins (as active versus *Candida* or *Staphylococcus aureus*). Thus, a serious lack of Th17 cells may lead to increased susceptibility to opportunistic infections and hyper-IgE syndrome.

### 4.2. Differentiation

The main cytokines that stimulate the Th17 differentiation are TGF*β*, IL-6, IL-21, and IL-23 (produced by dendritic cells and not involved in either Th1 or Th2 response) [41]. Cytokines produced by Th17 cells are IL-17 and IL-21 [42]. IL-17 has a pathogenetic role in both psoriasis and IBD. Some types of Th17 also produce IL-22 that has a pathogenetic role in psoriasis but a protective one in IBD. Some IL-22 producing cells do not secrete IL-17 and are called Th22; their differentiation is induced by IL-23, IL-6 ± TNF*α*.

What induces the Th17 differentiation seems to be the presence of TGF*β* and IL-6 in the microenvironment in which T-helper naïve cells stay. 

However, it is not clear if other elements drive the differentiation of Th17 cells. IL-23 is involved in the development of specific populations of Th17 cells, but this cytokine alone fails to induce differentiation of naïve T cells into Th17 [43]. IL-21 has been shown to initiate an alternative route for the activation of the Th17 population [44]. In humans, a combination of TGF-*β*, IL-1*β*, and IL-23 stimulates differentiation of Th17 cells starting from naive T cells [41]. Both the IFN*γ* and IL-4, the main stimulators of differentiation into Th1 and Th2, have been shown to negatively regulate the differentiation into Th17.

### 4.3. Functions

Th17 cells have an important role in many autoimmune diseases and infections (those involving *Propionibacterium acnes*, *Citrobacter rodentium*, *Klebsiella pneumoniae*, *Bacteroides*, *Borrelia*, *Mycobacterium tuberculosis*, and *Candida albicans*) [45–48]. The IL-17 that they produce promotes the chemotaxis of neutrophils and monocytes and the migration and activation of T lymphocytes and neoangiogenesis; it also induces the production of other cytokines (such as IL-6, GCSF, GMCSF, IL-1*β*, TGF, and TNF*α*), chemokines (including IL-8, GRO-*α*, and MCP-1), and prostaglandins (e.g., PGE2) by different cells (fibroblasts, endothelial cells, epithelial cells, keratinocytes, and macrophages).

### 4.4. Interleukin 23 and Th17

IL-23 is a heterodimeric cytokine composed of two subunits: the subunit *α* (p19) is encoded by the IL-23 gene in humans while subunit (p40) is shared with the IL-12; [49]. 

The IL-23 receptor consists of the subunit *β* of IL-12R (IL-12RB1) and a specific subunit, the IL-23R. Both IL-23 and IL-12 can activate the transcription factor STAT4 and stimulate the production of IFN-*γ*. Unlike IL-12, which acts primarily on naive CD4 T cells, IL-23 acts preferentially on memory CD4 T cells. IL-23 plays an important role in the inflammatory response against infections, promotes the upregulation of the matrix metalloprotease MMP9, increases angiogenesis, and reduces the infiltration of CD8 T cells. In collaboration with IL-6 and TGF-*β*1, IL-23 stimulates CD4 T cells to differentiate into Th17 cells.

### 4.5. Th17 in IBD

An alteration of Th17 has been suggested in IBD since increased levels of IL-23 and IL-17 have been found in the inflamed mucosa of patients suffering from these diseases [50]. 

### 4.6. Th17 in Skin Diseases

Th17 cells promote acanthosis, hyperkeratosis, and parakeratosis as well as the synthesis of inflammatory molecules within dermis and epidermis [51, 52]. In patients suffering for psoriasis, biopsies of injured skin show a high number of Th17 and high levels of TGF-*β*1, IL-6, IL-15, IL-17, IL-22, and IL-23 [53, 54]. Apart from psoriasis, Th17 cells have been related to other inflammatory skin diseases such as allergic contact dermatitis, atopic dermatitis, systemic sclerosis, and Behcet's disease [55, 56].

### 4.7. T Regulatory Cells (T-Regs)

The regulatory T cells (T-reg, also called T-suppressor) are a subpopulation of T cells specialized in suppressing the abnormal activation of the immune system in order to avoid excessive responses and to preserve the tolerance to self in different organs, including intestine and skin [57]. The interest for these cells has increased in recent years in relation to their possible use in the treatment of autoimmune diseases.

### 4.8. Populations of Regulatory T Cells

Regulatory T cells require the presence of IL-2 and TGF*β* to reach the full functionality [58]. These cells can be observed in various forms, including those CD8+ and those CD4+ CD25+ Foxp3+ (which are the “natural T-regs”) and other additional subpopulations, including Tr1, Th3, CD8+ CD28-, and T cells restricted to Qa-1. Abnormalities in the number and function of T-regs have been described in psoriasis and several other autoimmune diseases such as myasthenia gravis, Sjogren's syndrome, systemic lupus erythematosus, juvenile idiopathic arthritis, rheumatoid arthritis, and diabetes mellitus type I [59–64].

### 4.9. T-Regs in IBD

In the active phase of CD and UC the number of T-regs in peripheral blood is reduced if compared to controls; this is not observed during the remission phase of the diseases, suggesting that in the course of IBD T-regs migrate from peripheral blood to the inflamed intestinal mucosa, as it was demonstrated within lamina propria and mesenteric lymphnodes [65, 66].

### 4.10. T-Regs in Skin Diseases

An altered recruitment and/or function of T-regs can be an important pathogenetic factor in skin diseases, although the exact mechanisms are still unknown [67]. 

Functional and numerical abnormalities of T-regs have been also highlighted in the peripheral blood and skin lesions (especially in the superficial dermis) of patients with psoriasis [68]. 

### 4.11. The T-Regs and Th17 Relationship

The interesting dichotomous relationship between Th17 and T-regs has been postulated and defined by the discovery that TGF*β* is responsible for the induction of both lymphocyte subtypes. In fact TGF-*β* participates in the induction of T-regs, but in the presence of IL-6 it contributes to the induction of Th17 [69]. Recent evidences shows that, in presence of an inflammatory microenvironment, T-regs can convert into TH17, thus focusing on the importance of establishing the TH17/T-regs balance in human diseases [70, 71]. 

## 5. Conclusions

Psoriasis and IBD are strictly related inflammatory diseases, probably sharing immune-pathogenetic mechanisms. Skin and bowel represent, at the same time, barrier and connection between the inner and the outer sides of the body. This explains why, at these levels, immune processes play a key role in maintaining homeostasis and in sustaining pathological processes. 

The wide therapeutic overlaps between psoriasis and IBDs further support the hypothesis of a common pathogenesis.

## References

[1] Rudikoff D, Baral J, Lebwohl M (1999). Psoriasis and Crohn’s disease. *The Mount Sinai Journal of Medicine*.

[2] Hoffmann R, Schieferstein G, Schunter F, Jenss H (1991). Re: increased occurrence of psoriasis in patients with Crohn’s disease and their relatives. *The American Journal of Gastroenterology*.

[3] Potenza C, Annetta A, Bernardini N (2010). *Plaque Psoriasis: Anatomical, Clinical and Immunohistochemical Correlations During Anti-Tnfa Treatment*.

[4] Hammer B, Ashurst P, Naish J (1968). Diseases associated with ulcerative colitis and Crohn’s disease. *Gut*.

[5] Yamamoto T, Yokoyama A (1997). Coexistence of psoriasis vulgaris, ulcerative colitis, and thyroid adenoma. *Journal of Dermatology*.

[6] McEwen C, DiTata D, Lingg C, Porini A, Good A, Rankin T (1971). Ankylosing spondylitis and spondylitis accompanying ulcerative colitis, regional enteritis, psoriasis and Reiter’s disease. A comparative study. *Arthritis and Rheumatism*.

[7] Oestreicher J, Walters I, Kikuchi T, Gilleaudeau P, Surette J, Schwertschlag U (2001). Molecular classification of psoriasis disease-associated genes through pharmacogenomic expression profiling. *Pharmacogenomics Journal*.

[8] Farber EM, Nall ML, Watson W (1974). Natural history of psoriasis in 61 twin pairs. *Archives of Dermatology*.

[9] Friedman S, Blumberg RS, Braunwald E, Fauci AS, Kasper DL, Hauser SL, Longo DL, Jameson JL (2001). Inflammatory bowel disease. *Harrison’s Principles of Internal Medicine*.

[10] Yang H, Plevy SE, Taylor K (1999). Linkage of Crohn’s disease to the major histocompatibility complex region is detected by multiple non-parametric analyses. *Gut*.

[11] Watts DA, Satsangi J (2002). The genetic jigsaw of inflammatory bowel disease. *Gut*.

[12] Nair RP, Henseler T, Jenisch S (1997). Evidence for two psoriasis susceptibility loci (HLA and 17q) and two novel candidate regions (16q and 20p) by genome-wide scan. *Human Molecular Genetics*.

[13] Lee FI, Bellary SV, Francis C (1990). Increased occurrence of psoriasis in patients with Crohn’s disease and their relatives. *The American Journal of Gastroenterology*.

[14] Bernstein CN, Wajda A, Blanchard JF (2005). The clustering of other chronic inflammatory diseases in inflammatory bowel disease: a population-based study. *Gastroenterology*.

[15] Cohen AD, Dreiher J, Birkenfeld S (2009). Psoriasis associated with ulcerative colitis and Crohn’s disease. *Journal of the European Academy of Dermatology and Venereology*.

[16] Christophers E (2007). Comorbidities in psoriasis. *Clinics in Dermatology*.

[17] Burton PR, Clayton DG, Cardon LR (2007). Genome-wide association study of 14,000 cases of seven common diseases and 3,000 shared controls. *Nature*.

[18] Wolf N, Quaranta M, Prescott NJ (2008). Psoriasis is associated with pleiotropic susceptibility loci identified in type II diabetes and Crohn disease. *Journal of Medical Genetics*.

[19] Zeggini E, Weedon MN, Lindgren CM (2007). Replication of genome-wide association signals in UK samples reveals risk loci for type 2 diabetes. *Science*.

[20] Ogura Y, Bonen DK, Inohara N (2001). A frameshift mutation in NOD2 associated with susceptibility to Crohn’s disease. *Nature*.

[21] Bonen DK, Cho JH (2003). The genetics of inflammatory bowel disease. *Gastroenterology*.

[22] Lesage S, Zouali H, Cézard J-P (2002). CARD15/NOD2 mutational analysis and genotype-phenotype correlation in 612 patients with inflammatory bowel disease. *The American Journal of Human Genetics*.

[23] Inohara N, Nuñez G (2003). NODS: intracellular proteins involved in inflammation and apoptosis. *Nature Reviews Immunology*.

[24] Watanabe T, Kitani A, Murray PJ, Strober W (2004). NOD2 is a negative regulator of Toll-like receptor 2-mediated T helper type 1 responses. *Nature Immunology*.

[25] Nair RP, Stuart P, Ogura Y (2001). Lack of association between NOD2 3020insC frameshift mutation and psoriasis. *Journal of Investigative Dermatology*.

[26] Zhu K, Yin X, Tang X, Zhang F, Yang S, Zhang X (2012). Meta-analysis of NOD2/CARD15 polymorphisms with psoriasis and psoriatic arthritis. *Rheumatology International*.

[27] Yang S-K, Loftus EV, Sandborn WJ (2001). Epidemiology of inflammatory bowel disease in Asia. *Inflammatory Bowel Diseases*.

[28] Cargill M, Schrodi SJ, Chang M (2007). A large-scale genetic association study confirms IL12B and leads to the identification of IL23R as psoriasis-risk genes. *The American Journal of Human Genetics*.

[29] Smith RL, Warren RB, Eyre S (2008). Polymorphisms in the IL-12*β*and IL-23R genes are associated with psoriasis of early onset in a UK cohort. *Journal of Investigative Dermatology*.

[30] Capon F, di Meglio P, Szaub J (2007). Sequence variants in the genes for the interleukin-23 receptor (IL23R) and its ligand (IL12B) confer protection against psoriasis. *Human Genetics*.

[31] Nair RP, Ruether A, Stuart PE (2008). Polymorphisms of the IL12B and IL23R genes are associated with psoriasis. *Journal of Investigative Dermatology*.

[32] Chandran V (2010). Genetics of psoriasis and psoriatic arthritis. *The Indian Journal of Dermatology*.

[33] Nair RP, Stuart PE, Nistor I (2006). Sequence and haplotype analysis supports HLA-C as the psoriasis susceptibility 1 gene. *The American Journal of Human Genetics*.

[34] Gudjonsson JE, Karason A, Runarsdottir EH (2006). Distinct clinical differences between HLA-Cw*0602 positive and negative psoriasis patients—an analysis of 1019 HLA-C- and HLA-B-typed patients. *Journal of Investigative Dermatology*.

[35] Najarian DJ, Gottlieb AB (2003). Connections between psoriasis and Crohn’s disease. *Journal of the American Academy of Dermatology*.

[36] Friberg C, Björck K, Nilsson S, Inerot A, Wahlström J, Samuelsson L (2006). Analysis of chromosome 5q31-32 and psoriasis: confirmation of a susceptibility locus but no association with SNPs within SLC22A4 and SLC22A5. *Journal of Investigative Dermatology*.

[37] Li Y, Chang M, Schrodi SJ (2008). The 5q31 variants associated with psoriasis and Crohn’s disease are distinct. *Human Molecular Genetics*.

[38] Chang M, Li Y, Yan C (2008). Variants in the 5q31 cytokine gene cluster are associated with psoriasis. *Genes and Immunity*.

[39] Stockinger B, Veldhoen M (2007). Differentiation and function of Th17 T cells. *Current Opinion in Immunology*.

[40] Steinman L (2007). A brief history of TH17, the first major revision in the TH1/TH2 hypothesis of T cell-mediated tissue damage. *Nature Medicine*.

[41] Manel N, Unutmaz D, Littman DR (2008). The differentiation of human TH-17 cells requires transforming growth factor-*β* and induction of the nuclear receptor ROR*γ*t. *Nature Immunology*.

[42] Ouyang W, Kolls JK, Zheng Y (2008). The biological functions of T helper 17 cell effector cytokines in inflammation. *Immunity*.

[43] Bettelli E, Carrier Y, Gao W (2006). Reciprocal developmental pathways for the generation of pathogenic effector TH17 and regulatory T cells. *Nature*.

[44] Korn T, Bettelli E, Gao W (2007). IL-21 initiates an alternative pathway to induce proinflammatory T H17 cells. *Nature*.

[45] Ye P, Garvey PB, Zhang P (2001). Interleukin-17 and lung host defense against klebsiella pneumoniae infection. *The American Journal of Respiratory Cell and Molecular Biology*.

[46] Infante-Duarte C, Horton HF, Byrne MC, Kamradt T (2000). Microbial lipopeptides induce the production of IL-17 in Th cells. *Journal of Immunology*.

[47] Khader SA, Cooper AM (2008). IL-23 and IL-17 in tuberculosis. *Cytokine*.

[48] Huang W, Na L, Fidel PL, Schwarzenberger P (2004). Requirement of interleukin-17A for systemic anti-Candida albicans host defense in mice. *Journal of Infectious Diseases*.

[49] Oppmann B, Lesley R, Blom B (2000). Novel p19 protein engages IL-12p40 to form a cytokine, IL-23, with biological activities similar as well as distinct from IL-12. *Immunity*.

[50] Fujino S, Andoh A, Bamba S (2003). Increased expression of interleukin 17 in inflammatory bowel disease. *Gut*.

[51] Fitch E, Harper E, Skorcheva I, Kurtz SE, Blauvelt A (2007). Pathophysiology of psoriasis: recent advances on IL-23 and TH17 cytokines. *Current Rheumatology Reports*.

[52] Asarch A, Barak O, Loo DS, Gottlieb AB (2008). Th17 cells: a new paradigm for cutaneous inflammation. *Journal of Dermatological Treatment*.

[53] Lee E, Trepicchio WL, Oestreicher JL (2004). Increased expression of interleukin 23 p19 and p40 in lesional skin of patients with psoriasis vulgaris. *Journal of Experimental Medicine*.

[54] Sabat R, Philipp S, Höflich C (2007). Immunopathogenesis of psoriasis. *Experimental Dermatology*.

[55] Wolk K, Witte E, Wallace E (2006). IL-22 regulates the expression of genes responsible for antimicrobial defense, cellular differentiation, and mobility in keratinocytes: a potential role in psoriasis. *The European Journal of Immunology*.

[56] Toda M, Leung DYM, Molet S (2003). Polarized in vivo expression of IL-11 and IL-17 between acute and chronic skin lesions. *Journal of Allergy and Clinical Immunology*.

[57] Sakaguchi S, Powrie F (2007). Emerging challenges in regulatory T cell function and biology. *Science*.

[58] Zhang L, Zhao Y (2007). The regulation of Foxp3 expression in regulatory CD4^+^CD25^+^T cells: multiple pathways on the road. *Journal of Cellular Physiology*.

[59] Fattorossi A, Battaglia A, Buzzonetti A, Ciaraffa F, Scambia G, Evoli A (2005). Circulating and thymic CD4^+^ CD25^+^ T regulatory cells in myasthenia gravis: effect of immunosuppressive treatment. *Immunology*.

[60] Liu M-F, Lin L-H, Weng C-T, Weng M-Y (2008). Decreased CD4+CD25+bright T cells in peripheral blood of patients with primary Sjögren’s syndrome. *Lupus*.

[61] Zhang B, Zhang X, Tang F, Zhu L, Liu Y (2008). Reduction of forkhead box P3 levels in CD4^+^CD25^high^ T cells in patients with new-onset systemic lupus erythematosus. *Clinical and Experimental Immunology*.

[62] Wei C-M, Lee J-H, Wang L-C, Yang Y-H, Chang L-Y, Chiang B-L (2008). Frequency and phenotypic analysis of CD4+CD25+ regulatory T cells in children with juvenile idiopathic arthritis. *Journal of Microbiology, Immunology and Infection*.

[63] Ehrenstein MR, Evans JG, Singh A (2004). Compromised function of regulatory T cells in rheumatoid arthritis and reversal by anti-TNF*α* therapy. *Journal of Experimental Medicine*.

[64] Lindley S, Dayan CM, Bishop A, Roep BO, Peatman M, Tree TIM (2005). Defective suppressor function in CD4^+^CD25^+^ T-cells from patients with type 1 diabetes. *Diabetes*.

[65] Saruta M, Yu QT, Fleshner PR (2007). Characterization of FOXP3^+^CD4^+^ regulatory T cells in Crohn’s disease. *Clinical Immunology*.

[66] Yu QT, Saruta M, Avanesyan A, Fleshner PR, Banham AH, Papadakis KA (2007). Expression and functional characterization of FOXP3^+^CD4^+^ regulatory T cells in ulcerative colitis. *Inflammatory Bowel Diseases*.

[67] Hirahara K, Liu L, Clark RA, Yamanaka K-I, Fuhlbrigge RC, Kupper TS (2006). The majority of human peripheral blood CD4+CD25 highFoxp3+ regulatory T cells bear functional skin-homing receptors. *Journal of Immunology*.

[68] Bovenschen HJ, van Vlijmen-Willems IMJJ, van de Kerkhof PCM, van Erp PEJ (2006). Identification of lesional CD4+ CD25+ Foxp3+ regulatory T cells in psoriasis. *Dermatology*.

[69] Bettelli E, Carrier Y, Gao W (2006). Reciprocal developmental pathways for the generation of pathogenic effector TH17 and regulatory T cells. *Nature*.

[70] Radhakrishnan S, Cabrera R, Schenk EL (2008). Reprogrammed FoxP3+ T regulatory cells become IL-17+ antigen-specific autoimmune effectors in vitro and in vivo. *Journal of Immunology*.

[71] Oukka M (2007). Interplay between pathogenic Th17 and regulatory T cells. *Annals of the Rheumatic Diseases*.

